# Comparing sedation protocols for endoscopic retrograde cholangiopancreatography (ERCP): A retrospective study

**DOI:** 10.1016/j.heliyon.2024.e27447

**Published:** 2024-03-01

**Authors:** Ning Zhang, Guanjun Li

**Affiliations:** a Department of Cardiopulmonary Rehabilitation, Shandong Provincial Third Hospital, No.12, Wuyingshan Middle Road, Jinan, Shandong, 250000, China; b Department of Anesthesiology, Shandong Provincial Third Hospital, No.12, Wuyingshan Middle Road, Jinan, Shandong, 250000, China

**Keywords:** Adverse events, Dexmedetomidine, Endoscopic retrograde cholangiopancreatography (ERCP), Ketamine, Propofol, Sedation

## Abstract

**Background:**

Endoscopic retrograde cholangiopancreatography (ERCP) is a widely used diagnostic and therapeutic procedure. Effective sedation is crucial to enhance patient comfort and optimize endoscopist performance. Various sedation protocols, including Propofol and Dexmedetomidine (Pro-Dex), Ketamine and Propofol (Keto-Fol), Propofol and Midazolam (Pro-Mid), and Propofol alone, have been utilized during ERCP. This retrospective study aims to compare the safety and efficacy of these four sedation protocols.

**Methods:**

A retrospective analysis was conducted on data from 600 patients who underwent ERCP between 2018 and 2021, with each patient receiving one of the four sedation protocols. Protocol assignment was based on the endoscopist's preference. Data on hemodynamic parameters, sedation level, recovery time, and procedure-related complications were collected.

**Results:**

Baseline data showed no significant differences among the groups pre-procedure. The Pro-Dex group exhibited significantly lower mean total propofol dose, shorter recovery time, and faster achievement of Ramsay Sedation Scale (RSS) score 3–4 compared to the other groups. The Pro-group demonstrated significantly longer hospital stay than the other three groups (median, 4.19 ± 1.1 vs. 3.48 ± 1.2 days in the KP groups, p = 0.042). There were no significant variations in the incidence of respiratory depression, hypotension, or bradycardia among the four groups. Additionally, notable trends were found for hemodynamic measures, total propofol dosage, time to reach the desired level of sedation (as measured by the Ramsay Sedation Scale), and hospital stay based on BMI categories, indicating that higher BMI is linked to more serious outcomes.

**Conclusion:**

Our retrospective study demonstrates that the Pro-Dex protocol offers superior sedation quality, faster recovery, and fewer complications compared to the other protocols during ERCP. However, the incidence of ERCP-related adverse events did not significantly differ among the four sedation protocols. These findings can aid clinicians in selecting the most appropriate sedation protocol for ERCP, considering patient and endoscopist preferences.

## 1 Introduction

Endoscopic retrograde cholangiopancreatography (ERCP) is a minimally invasive endoscopic procedure that combines fluoroscopy and endoscopy to visualize the biliary and pancreatic ducts and to perform therapeutic interventions [1]. Here there were many challenges during sedation for ERCP in the endoscopic unit, such as remote location, semi-prone position, less familiar area, long procedure and common airway [2]. It should ensure immobility, prevent coughing or wheezing, adequate analgesia, and allow the patient to avoid any complications such as perforation or peritonitis. The procedure is typically performed under moderate-to-deep sedation to ensure patient comfort and safety [3]. There are a number of different sedative agents that can be used for ERCP, including propofol, dexmedetomidine, ketamine, and midazolam [4]. The choice of sedative agent depends on a number of factors, such as the patient's age, medical history, and the anticipated duration of the procedure [5,6].

Propofol is a short-acting sedative that is often used for ERCP because it provides rapid onset and recovery of sedation. Its analgesic properties are limited, and additional analgesia may be required. Moreover, lead to analgesics and minor adverse effects, including transient hypotension, respiratory depression, and hypoventilation [7]. Dexmedetomidine a selective α-2 agonist with sedative and analgesic properties, and its most important advantage is that it does not cause respiratory depression [8]. Therefore, today, its premedication is increasing. Ketamine is an anesthetic agent that can be used for ERCP to provide deep sedation and analgesia [9]. Midazolam is a benzodiazepine that is often used in combination with other sedative agents to provide additional anxiolysis [10].

The optimal sedative agent for ERCP is still being debated. Some studies have recommended that propofol alone may be appropriate for most patients [11], However, other studies have found that the addition of dexmedetomidine or ketamine can improve patient satisfaction and reduce the risk of complications [9,12]. In this regard, the results of some recent reports have indicated that the combination of sedative medicines such as propofol, dexmedetomidine, and ketamine can adverse events. The combination of these sedatives may also help to neutralize the adverse effects of each other, resulting in the best and most effective sedation with the least adverse effects for the patient. For example, a study by Inatomi et al. (2018) found that propofol with dexmedetomidine was associated with a lower incidence of adverse events and a higher patient satisfaction score than propofol alone in patients undergoing ERCP [13]. Another study by Yin et al. (2019) found that propofol with ketamine was associated with a shorter procedure time and a lower incidence of adverse events than propofol alone in patients undergoing ERCP [14]. However, other studies have found no significant difference in the efficacy or safety of different sedative regimens for ERCP [15]. For example, a study by Gary Andolfatto et al. (2012) found that Ketofol was not significantly different from propofol alone in terms of the incidence of adverse events or patient satisfaction in patients undergoing ERCP [15]. In addition to the studies mentioned above, there have been a number of other studies that have compared the safety and efficacy of different sedative regimens for ERCP. The results of these studies have been mixed, with some studies finding that one sedative regimen is superior to another, and other studies finding no significant difference between the regimens.

The lack of a standard sedative approach for ERCP procedures has led to a need for further research in this area. While numerous studies have compared the sedative effects and potential side effects of various drugs, no studies have examined the combined effects of four different sedative regimens during ERCP procedures. As a result, this retrospective study aimed to compare the safety and efficacy of four sedative regimens for ERCP: Propofol and Dexmedetomidine (Pro-Dex), Ketamine and Propofol (Keto-Fol), Propofol and Midazolam (Pro-Mid) and Propofol Alone. We hypothesized that a subdissociative dose of ketamine or dexmedetomidine is safer than the propofol alone for sedation during ERCP. To this end, we compared the patient data, procedure duration, hemodynamic variables, Ramsay Sedation Score (RSS), recovery time, and complications of each groups.

## 2 Methods

### 2.1 Study design

This study was performed in accordance with the STROBE recommendations for reporting observational studies in epidemiology. This study was conducted according to the guidelines of the Declaration of Helsinki. The Institutional Review Board of Shandong University approved this study (KYLL-2021088) and waived the requirement of informed consent due to the retrospective nature of this study and its observational design.

### 2.2 Sample size

Drawing upon findings from a previous study [16], the sample size for the current research was established to adequately achieve an 80% probability of detecting clinically meaningful reductions in HR and MAP fluctuations by 10–20%, particularly post-anesthesia induction and intubation. The Student's t-test for independent samples was employed for the calculation, with a significance level of 0.05 and a power of 80%. The final sample size was determined to be 600 participants. Each group was assumed to have an equal number of participants, with 150 individuals in each group. The calculations were completed using PS Power and Sample Size Calculations Software, version 3.0.11.

**Fig. 1:**
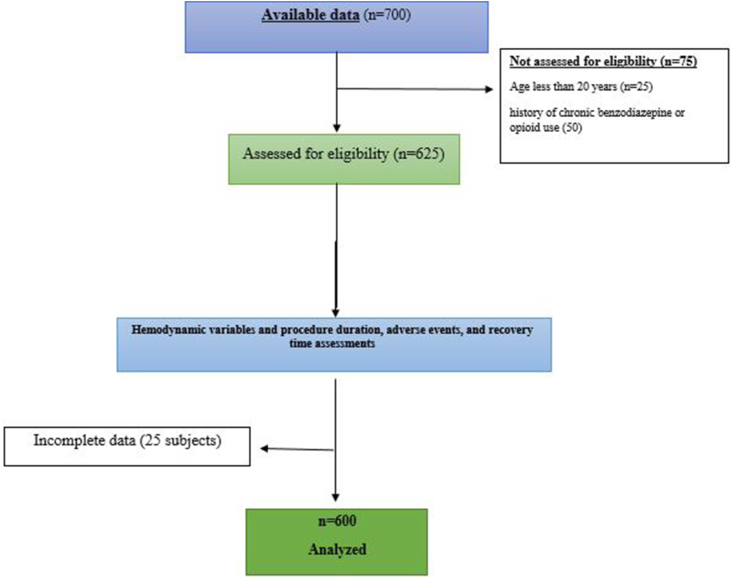
Flow diagram displaying the progress of all participants through the study.

We conducted a retrospective review of patient records from a tertiary care hospital. Six hundred patients were included in this study. Between June 2018 and September 2021, patients underwent ERCP procedures at Shandong Provincial Third Hospital Medical Center, a tertiary referral hospital in Shandong, China. All procedures were performed by four experienced endoscopists who each had performed more than 2000 ERCP examinations. All authors had access to the study data and had reviewed and approved the final manuscript. All patients were graded according to the American Society of Anesthesiologists (ASA) physical status classification system. Patients were selected using electronic medical records and our endoscopy database. We assessed the following characteristics: patient demographic information (age, sex), body mass index (kg/m2), pre-procedure ASA classification, ERCP indications, and duration of the procedure (time interval from insertion to final withdrawal of the endoscope). Anesthesia records were accessed to record the total dose of sedatives (mg). The data was collected by two independent reviewers to ensure accuracy. Electronic records of sedation-related adverse events (SAEs) were maintained by the nurse anesthetist. Patients who were less than 20 years old was excluded from this study. If they were graded as ASA class III to V. if they had mental disorders; or if they had a history of chronic benzodiazepine or opioid use. During this study, each patient participated only once, as depicted in Fig. 1, which is a visual representation of the study's design. A total of 150 individuals were chosen for each of the four sedative regimens.

### 2.3 Anesthetic procedure

Patients underwent a fasting period of at least 6 h before the ERCP procedure. Following the establishment of intravenous access, a 500 mL infusion of Ringer's solution was initiated at a rate of 250 mL/h. Patients were positioned semi-prone and received 2L/min of oxygen through a nasal cannula. To enhance comfort during the procedure, patients were given topical anesthesia to the laryngopharynx using 2% lidocaine. Additionally, they received a premedication dose of 0.2 μg/kg sufentanil intravenously. In the Propofol group, patients were administered an initial bolus of 1–2 mg/kg propofol over 30 s, followed by a propofol infusion at 2–3 mg/kg/h. Patients in the Pro-Dex group were given a bolus of dexmedetomidine at 1 μg/kg and propofol at 2 mg/kg over 10 min, followed by an infusion of dexmedetomidine at 1.2 μg/kg/h and propofol at 4–6 mg/kg/h. Patients in the Keto-Fol group received a bolus of ketamine at 1 mg/kg and propofol at 2 mg/kg over 10 min, followed by an infusion of ketamine at 0.5 mg/kg/h and propofol at 4–6 mg/kg/h. Patients in the Pro-Mid group received a standard dose of 2.5 mg midazolam at the beginning of the procedure, followed by a loading dose of propofol adjusted to patient weight immediately after the midazolam application.

### 2.4 Monitoring the patient during the procedures

The patient's heart rate (HR), systolic blood pressure (SBP), diastolic blood pressure (DBP), peripheral oxygen saturation (SPO2), and mean arterial pressure (MAP) were continuously monitored by the nurse anesthetist during the procedure. All vitals were recorded at baseline, just before the start of sedation and every 5 min during the procedure. If there was any sign of apnea-hypopnea by nasal capnography and clinical observation for more than 10 s, the patient was evaluated by the nurse anesthetist and airway manipulation (jaw thrust, chin lift, or nasal airway placement) was performed if needed. If SpO2 falls below 90% for more than 30 s with supplemental oxygen and jaw pressure, the procedure is stopped until normal oxygen saturation is achieved. If the patient had apnea, it was triggered by noxious stimuli. When this was insufficient, the endoscope was removed and bag-mask assisted ventilation was performed, followed by endotracheal intubation if clinically necessary.

### 2.5 Procedure assessments

We assessed the time to initial sedation, total dosage of sedatives during the procedure, and total procedure duration. We defined deep sedation as a modified Observer's Assessment of Alertness/Sedation (OAA/S) score of 2, which is the state at which the patient can respond, but only after mild prodding or shaking. We recorded the time it took for the patient to reach deep sedation after receiving the initial sedative administration. We attempted to maintain patients at a modified OAA/S score of 2 throughout the procedure. We also recorded the total dosage of sedatives and the total procedure duration.

### 2.6 Study outcomes

The primary outcome was the incidence of adverse events during or after the procedure. Secondary outcomes included procedure time, Total dose of propofol, recovery time, and Parameters of Sedation Efficacy, Sedation-related adverse events including Apnea, Nausea/Vomiting, Desaturation, Hallucination or excitation, Hypotension, oxygen saturation, Heart rate, and blood pressure and Bradycardia. If the pulse oximeter oxygen saturation (SpO2) declined below 90%, we supplied oxygen by nasal cannula at 6 L/min until SpO2 recovered. An SpO2 of less than 90% for more than 10 s was defined as a desaturation event. Changes in blood pressure greater than 20% of the baseline value and changes in the pulse rate greater than 50% were also considered significant adverse events, specifically bradycardia. In addition, 30-day mortality, surgical complications, bleeding, perforation, and infection were assessed.

### 2.7 Recovery

Recovery after sedation was assessed using the modified Aldrete score, which was measured every 5 min after the endoscope was removed (i.e., at 5, 10, and 15 min). The modified Aldrete score is a post-anesthesia recovery score that takes into account five criteria: activity, respiration, circulation, consciousness, and oxygen saturation. A score of 0, 1, or 2 is given for each criterion, with 2 being the ideal condition. A score of 9 or more indicates that the patient is ready to be transferred from the recovery area to the inpatient ward.

### 2.8 Data analysis

The normality of the continuous variables was assessed using the Kolmogorov-Smirnov test. If the distribution was normal, the values were presented as mean ± standard deviation. If the distribution was not normal, the values were presented as medians and ranges. Categorical outcomes were analyzed by the chi-square test or Fisher exact tests, when appropriate. Continuous outcomes were analyzed with Student t-test for normally distributed data. A p-value of <0.05 was considered statistically significant. When comparing 4 means, the ANOVA test was applied. In this study, we categorized all patients regardless of their medication into three groups based on both BMI and age, with the aim of comparing all outcomes across these different categories. It was considered highly significant when the *P*-value was 0.01. All statistical analyses were performed using SPSS software version 18.0 (IBM Co, USA).

## 3 Results

Patient characteristics and ERCP procedure details are shown in Table 1. As presented, 600 patients were included, out of which 189 were men and 411 women which impales 31.5 in men, and the mean age was 59.16 years. There were no significant differences between the groups with regard to age, sex, BMI, ASA classification, total procedure duration, and baseline cardiorespiratory parameters including SBP, DBP, and MAP.

**Table 1:** Table 1 Patient characteristics across anesthesia types.

Variables	PRO Group (n = 150)	MP group (n = 150)	DP group (n = 150)	KP group (n = 150)	P value
**Age** (years)	65.86 ± 12.72	63.53 ± 10.30	60.90 ± 11.90	63.01 ± 11.73	0.004
**Gender**, Male/Female	47/103	49/101	43/107	50/100	0.828
**BMI** (kg/m)	28.8 ± 1.86	28.42 ± 3.29	28.78 ± 2.4	28.3 ± 5.14	0.985
**ASA** classification, no. (%)					0.932
І	38 (25)	45 (30)	41 (27)	38 (25)	
ІІ	74 (50)	69 (47)	69 (47)	77 (51)	
ІІІ	38 (25)	36 (23)	40 (26)	35 (24)	
**Cigarette smoking** n (%)	22 (14.7)	29 (19.3)	30 (20)	23 (15.3)	0.508
**Alcohol intake** n (%)	26 (17.3)	21 (14)	27 (18)	18 (12)	0.428
**Comorbidities** n (%)					0.156
**Cardiovascular disease**	30 (20)	35 (23)	40 (26)	41 (27)	
**Respiratory disease**	3 (2)	5 (3)	5 (3)	10 (6)	
**Diabetes**	12 (8)	18 (12)	13 (8.5)	15 (9)	
**ERCP indications**					0.843
Bile duct stone	31 (2.7)	37 (24.7)	41 (27.3)	42 (28)	
Benign obstruction	98 (65.3)	93 (62)	91 (60.7)	89 (59.3)	
Malignant obstruction	21 (14)	20 (13.3)	18 (12)	19 (12.7)	
Time of the procedure (min)	36.39 ± 3.2	37.18 ± 3.9	35.92 ± 4.1	29.1 ± 3.7	0.289
Baseline **SBP**(mmHg)	118.54 ± 12.2	115.95 ± 15.3	125.17 ± 13.21	121.91 ± 15.63	0.150
Baseline **DBP**(mm/Hg)	82.33 ± 11.08	77.52 ± 12.84	81.58 ± 12.9	82.35 ± 14.03	0.761
Baseline **MAP** (mmHg)	99.4 ± 14.2	102.3 ± 15.2	98.5 ± 13.1	101.6 ± 11.1	0.051

Abbreviations: PRO: Propofol, DP: dexmedetomidine plus Propofol, MP, Midazolam plus Propofol, KP, Ketamine plus Propofol.

### 3.1 Drug dosage and parameters of sedation efficacy

Table 2 showed total dose of propofol, recovery time, and parameters of sedation efficacy in the four studied groups. Patients in the DP group achieved desired RSS of 3–4 more rapidly, which allowed for earlier endoscopic procedures (8.3 min [DP group] vs. 12.22 min [PRO group]; p < 0.001). Total propofol consumption was compared between groups. The cumulative of propofol dose required in the DP group was 180.2 ± 50.2 mg which less than other groups (p < 0.001). In addition, patients in the DP group had decreased duration required for recovery when compared with those in other groups (P = 0.001).

**Table 2:** Total dose of propofol, recovery time, and Parameters of Sedation Efficacy in the Four studied groups.

Variables	PRO Group (n = 150)	MP group (n = 150)	DP group (n = 150)	KP group (n = 150)	P value
Total dose of propofol (mg)	240.4 ± 36.1	216.1 ± 45.7	180.2 ± 50.2	190.2 ± 38.7	**0.002**
Recovery time (min)	26.38 ± 38.7	26.8 ± 45.7	9.95 ± 50.2	27.76 ± 36.1	**0.001**
Time to achieve desired RSS of 3–4	12.22 ± 3.3	10.02 ± 3.8	8.3 ± 3.1	12.04 ± 4.2	**0.001**
Time to achieve an Aldrete Recovery Scale Score of 9–10	10.28 ± 2.7	8.08 ± 3.7	6.4 ± 2.3	10.1 ± 2.2	**0.001**

Abbreviations: PRO: Propofol, DP: dexmedetomidine plus Propofol, MP, Midazolam plus Propofol, KP, Ketamine plus Propofol.

### 3.2 Sedation-related adverse events

The desaturation (SpO_2_ of less than 90%) was observed in 15 patients in PRO group, 9 patients in the MP, 6 In DP group and 9 in KP group, which no significant differences between groups (p = 0.196). There was no significant difference between the four groups with regard to events of Apnea, Hypotension, and Bradycardia. Nausea/Vomiting and Hallucination or excitation events are more frequent in KP group than other groups (p = 0.003 and p < 0.001, respectively). (Table 3).

**Table 3:** Sedation-related adverse events in the Four studied groups.

Variables	PRO Group (n = 150)	MP group (n = 150)	DP group (n = 150)	KP group (n = 150)	P value
Apnea	10 (6.7)	14 (9.3)	6 (4)	8 (5.3)	0.269
Nausea/Vomiting	10 (6.7)	9 (6)	6 (4)	22 (14.7)	**0.003**
Desaturation	15 (10)	9 (6)	6 (4)	9 (6)	0.196
Hallucination or excitation	0 (0)	0 (0)	0 (0)	12 (8)	**0.001**
Hypotension	15 (10)	7 (4.7)	6 (4)	7 (4.7)	0.094
Bradycardia	11 (7)	4 (2.7)	6 (4)	3 (2)	0.086

Abbreviations: PRO: Propofol, DP: dexmedetomidine plus Propofol, MP, Midazolam plus Propofol, KP, Ketamine plus Propofol.

The data are presented as number (%).

**Fig. 2:**
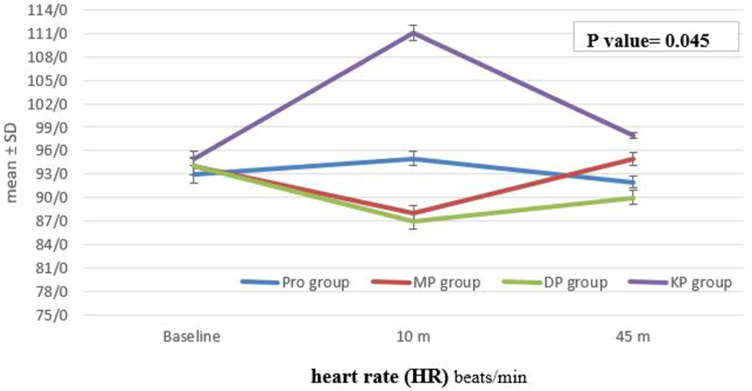
This figure compares the intra-procedural heart rate (**HR**) of the patients in the four groups at different time points. The boxplots show the median HR values for each group. A one-way ANOVA test was performed to compare HR between groups, with a statistically significant difference found between the groups (p = 0.045).

**Fig. 3:**
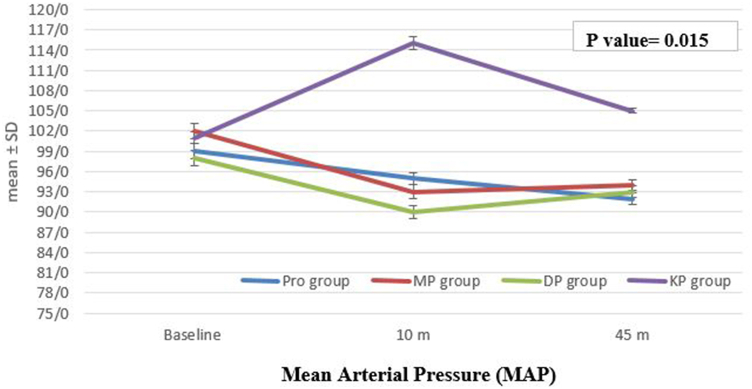
This figure compares Intra-Procedural Mean Arterial Pressure (**MAP**) of the patients in the four groups at different time points. The boxplots show the median MAP values for each group. A one-way ANOVA test was performed to compare MAP between groups, with a statistically significant difference found between the groups (p = 0.015).

**Fig. 4:**
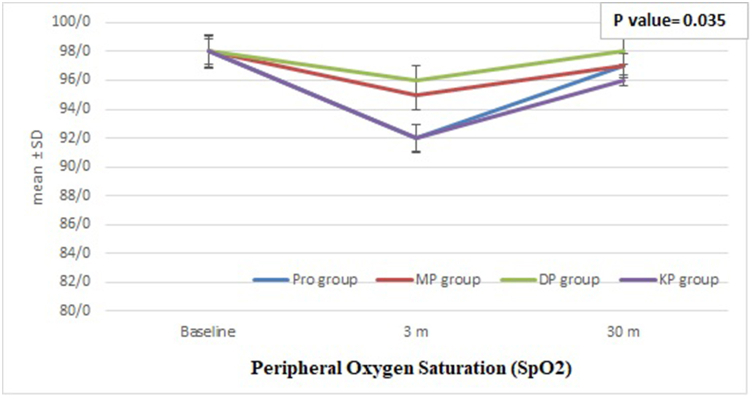
Comparison between Groups Regarding Intra-Procedural Peripheral Oxygen Saturation (**SpO2**). The boxplots illustrate the median values for each group. A one-way ANOVA test was performed to compare SpO2between groups, with a statistically significant difference found between the groups (p = 0.035).

As indicated in Fig. 2, Fig. 3, and Fig. 4 HR, MAP and SPO2 were comparable at baseline and no differences were observed, However Post-procedural HR changes demonstrated significant differences between four groups across the baseline, 10-min, and 45-min; HR values were lower in DP group and increased in KP groups (p = 0.045) [Fig. 2]. The intra-procedural HR decreased significantly in DP group, while it increased significantly in KP group from baseline till the 10 min procedures.

The intra-procedural MAP decreased significantly in all group from measuring at baseline till 10 min of the procedure, while it increased significantly in KP group (p < 0.01) from baseline till the 10 of the procedure (p = 0.015) [Fig. 3].

The intra-procedural SPO2 decreased significantly in all group from measuring at baseline till 10 min of the procedure, with more decreasing in PRO group (p = 0.035), But later it returned to near normal level in all groups [Fig. 4].

Table 4 presented major ERCP-related adverse events in the four studied groups. The most remarkable result to emerge from the data is that patients who received PRO had a significant longer hospital length of stay than those other groups (median, 4.19 ± 1.1 vs 3.48 ± 1.2 days in KP groups, p = 0.042). In Group PRO, five patients (3.4%) had bleeding and three patients (2 %) perforation. In Group MP, four patients (2.7%) had bleeding, ten patients (7%) had infection, and four patients (2.4%) had PEP. Nine patients (6.3%) had PEP, six (4%) bradycardia, Group DP. Also, seven patients (5%) had PEP, three patients (2%) bleeding in KP group. Patients in the PRO group exhibited a higher infection, perforation and bleeding, while patients DP group exhibited a higher PEP when compared to other groups, although not reached to significant levels (p > 0.05).

**Table 4:** ERCP-related adverse events in the four studied groups.

Variables	PRO Group	MP group	DP group	KP group	P value
30-day mortality	2 (1.3)	1 (0.7)	0 (0)	0 (0)	0.298
Adverse discharge, n	19 (12.7)	14 (9.3)	9 (6)	11 (7.3)	0.195
Hospital Stay in days, mean (SD)	4.19 ± 1.1	3.59 ± 0.7	3.76 ± 0.59	3.48 ± 1.2	**0.042**
Surgical complication					
Bleeding	5 (3.4)	4 (2.7)	3 (2)	3 (2)	0.565
Perforation	3 (2)	2 (1.4)	2 (1.4)	1 (1)	0.156
Infection	12 (8.5)	10 (7)	6 (4)	3 (2)	0.055
PEP	8 (5.5)	4 (2.4)	9 (6.3)	7 (5)	0.085

Abbreviations: PRO: Propofol, DP: Dexmedetomidine plus Propofol, MP, Midazolam plus Propofol, KP, Ketamine plus Propofol, PEP, Postendoscopic retrograde cholangiopancreatography pancreatitis; ERCP, Endoscopic retrograde cholangiopancreatography.

The data are presented as number (percent).

In this study, we examined the correlation between BMI category and age with the severity of outcomes. Table 5 displays the patient characteristics, measures of sedation effectiveness, sedation-related adverse events, and ERCP-related adverse events based on BMI. A significant trend was observed for SBP, DBP, MAP, total dose of propofol, time to achieve desired RSS of 3–4, time to achieve an Aldreth Recovery Scale Score of 9–10, and hospital stay across BMI categories. Our findings suggest that higher BMI is associated with more severe outcomes. However, we did not find any significant association between BMI and time of the procedure, recovery time, apnea, nausea/vomiting, desaturation, hallucination or excitation, hypotension, or bradycardia across BMI categories.

**Table 5:** Patient characteristics, parameters of sedation efficacy, sedation-related adverse events and ERCP-related adverse events across body mass index.

Variables	Body Mass Index			
	Category 1 (n = 34) *BMI<25*	Category 2 (n = 476) *BMI = 25–30*	Category 2 (n = 90) *BMI>30*	*P*-Value
Baseline SBP(mmHg)	119.74 ± 7.13	125.06 ± 8.46	131.67 ± 8.41	0.001
Baseline DBP(mm/Hg)	75.15 ± 4.52	82.91 ± 16.56	70.11 ± 7.86	0.001
Baseline MAP (mmHg)	79.66 ± 8.13	97.25 ± 6.49	125.6 ± 17.49	0.001
Time of the procedure (min)	31.32 ± 2.03	33.33 ± 4.89	42.57 ± 6.30	0.143
Total dose of propofol (mg)	246.78 ± 51.17	195.24 ± 63.59	269.34 ± 87.43	0.001
Recovery time (min)	21.36 ± 4.59	21.26 ± 3.46	29.87 ± 7.84	0.081
Time to achieve desired RSS of 3–4	6.45 ± 2.19	10.60 ± 4.64	12.55 ± 3.91	0.001
Time to achieve an Aldreth Recovery Scale Score of 9–10	4.51 ± 1.19	8.86 ± 4.84	10.62 ± 3.91	0.001
Hospital Stay in days	3.57 ± 0.99	3.56 ± 0.92	4.88 ± 1.38	0.001
Apnea	10 (6.7)	14 (9.3)	14 (9.3)	0.150
Nausea/Vomiting	0 (0)	35 (5.8)	8 (1.3)	0.233
Desaturation	1 (0.2)	36 (6)	2 (0.3)	0.547
Hallucination or excitation	0 (0)	12 (2.5)	0 (0)	0.213
Hypotension	10 (6.7)	13 (9)	12 (8.5)	0.492
Bradycardia	6 (3)	18 (3)	0 (0)	0.001
30-day mortality	0 (0)	3 (0.6)	0 (0)	0.675
Adverse discharge, n	8 (1.3)	40 (6.7)	5 (0.8)	0.008

The values are presented as either mean ± standard deviation and number (%).

*P*-Value were calculated using the one-way ANOVA test *.

Table 6 presents patient characteristics, measures of sedation effectiveness, sedation-related, and ERCP-related adverse events based on age categories. No significant differences were observed for these variables across age categories, except for MAP, total dose of propofol, time to achieve a desired RASS score of 3–4, time to achieve an Aldreth Recovery Scale score of 9–10, and bradycardia.

**Table 6:** Patient characteristics, parameters of sedation efficacy, sedation-related and ERCP-related adverse events across Age.

Variables	Age			
	Category 1 (n = 93) *Age<49*	Category 2 (n = 132) *Age = 50-60*	Category 2 (n = 364) *Age>61*	*P*-Value
Baseline SBP(mmHg)	123.37 ± 19.05	125.98 ± 16.93	126.24 ± 19.01	0.407
Baseline DBP(mm/Hg)	77.57 ± 1.27	79.94 ± 8.44	81.6 ± 8.75	0.082
Baseline MAP (mmHg)	100.34 ± 12.16	97.83 ± 11.53	101.7 ± 15.90	0.028
Time of the procedure (min)	23.91 ± 10.4	36.12 ± 1.76	36.78 ± 4.45	0.029
Total dose of propofol (mg)	178.83 ± 11.39	192.93 ± 51.83	211.85 ± 55.65	0.000
Recovery time (min)	19.48 ± 1.26	22.07 ± 8.74	24.65 ± 3.26	0.200
Time to achieve desired RSS of 3–4	9.26 ± 4.11	9.75 ± 3.06	11.20 ± 3.6	0.001
Time to achieve an Aldreth Recovery Scale Score of 9–10	7.32 ± 1.11	7.81 ± 3.06	9.27 ± 3.06	0.001
Hospital Stay in days	3.74 ± 1.7	3.89 ± 1.57	3.68 ± 0.96	0.673
Apnea	4 (4.3)	5 (3.8)	27 (7.4)	0.256
Nausea/Vomiting	7 (1.2)	5 (0.8)	30 (5.1)	0.226
Desaturation	2 (2.2)	8 (6.1)	26 (7.1)	0.197
Hallucination or excitation	3 (0.5)	3 (0.5)	5 (0.8)	0.472
Hypotension	2 (2.2)	6 (4.5)	24 (6.8)	0.233
Bradycardia	7 (7.5)	0 (0)	12 (3.3)	0.010
30-day mortality	0 (0)	0 (0)	2 (0.3)	0.723
Adverse discharge	5 (5.4)	9 (6.8)	34 (9.3)	0.097

The values are presented as either mean ± standard deviation and number (%).

*P*-Value were calculated using the one-way ANOVA test *.

## 4 Discussion

The study was retrospective and included 600 patients comparing different sedation regimens during ERCP highlights the potential benefits of the Pro-Dex protocol in terms of faster achievement of the desired sedation level, lower propofol consumption, and shorter recovery time. Although differences in adverse events were not significant, there was a trend towards higher rates of specific adverse events in certain groups. Regarding the length of hospital stay, patients who received Propofol alone had a significantly longer duration compared to the Keto-Fol group. This finding suggests that the choice of sedation protocol may have an impact on post-procedural recovery and the need for extended hospitalization. The Propofol alone group also exhibited higher rates of infection, perforation, and bleeding, while the Pro-Dex group had a higher incidence of post-ERCP pancreatitis. However, these differences did not reach statistical significance, indicating the need for larger studies to draw definitive conclusions regarding safety outcomes.

Our findings revealed several important insights into the sedation protocols employed during ERCP. The patient characteristics and ERCP procedure details were well-balanced among the four groups, with no significant differences observed in age, sex, BMI, ASA classification, total procedure duration, and baseline cardiorespiratory parameters including SBP, DBP, and MAP, minimizing the potential influence of confounding variables on our results. This enhances the internal validity and reliability of our findings.

The findings of this study are consistent with the findings of previous studies that have compared the safety and efficacy of different sedative regimens for ERCP [5,8,9,12,14,15]. Previous studies have also investigated sedation regimens during ERCP. For example, a randomized controlled trial by Ong et al. compared propofol alone to propofol combined with midazolam for ERCP sedation. Their findings demonstrated that the combination of propofol and midazolam resulted in faster recovery times and higher patient satisfaction compared to propofol alone [17]. These results align with the Pro-Mid group in our study, which also showed comparable sedation efficacy to the Pro-Dex group but had longer recovery times.

Another study by Peprahet al. evaluated the use of propofol and ketamine for ERCP sedation. They reported that the combination of propofol and ketamine provided effective sedation with minimal adverse events [4]. However, our study found a higher incidence of adverse events, such as nausea/vomiting and hallucination or excitation, in the Keto-Fol group compared to the other groups. This discrepancy may be attributed to variations in patient populations, procedural techniques, or differences in the dosing and administration protocols of sedative agents.

However, this study is the first to compare four different sedative regimens in a large, retrospective cohort. Regarding sedation efficacy, the Pro-Dex group exhibited a faster attainment of the desired RSS score of 3–4, resulting in earlier initiation of endoscopic procedures compared to the Propofol alone group [8,12]. This finding is consistent with previous studies that have demonstrated the synergistic effect of combining propofol and dexmedetomidine in achieving optimal sedation levels for various procedures [12]. The mechanism underlying this enhanced sedative effect may involve the selective activation of α2-adrenergic receptors by dexmedetomidine, leading to sedation and analgesia [18]. Dexmedetomidine's unique pharmacodynamic profile, including its anxiolytic and sympatholytic properties, may contribute to its ability to enhance the sedative effects of propofol [19]. Additionally, the Pro-Dex group required a significantly lower cumulative dose of propofol, indicating a more efficient sedation protocol. These findings highlight the potential benefits of combining propofol with dexmedetomidine for achieving adequate sedation during ERCP. Recovery time was also significantly shorter in the Pro-Dex group compared to the other groups. This suggests that the use of dexmedetomidine in combination with propofol may lead to a more rapid recovery from sedation, which can contribute to improved patient throughput and satisfaction.

In terms of sedation-related adverse events, our results indicated no significant differences between the four groups regarding desaturation, apnea, hypotension, and bradycardia. These findings are consistent with previous studies that have reported the safety profiles of propofol-based sedation regimens during ERCP [4,7,12]. However, it is worth that the incidence of nausea/vomiting and hallucination or excitation events was higher in the Keto-Fol group compared to the other groups. This aligns with the known side effects of ketamine, including its potential to induce hallucinations and unpleasant emergence reactions [12]. The mechanism underlying these adverse events may involve ketamine's antagonistic effect on *N*-methyl-D-aspartate (NMDA) receptors, leading to dissociative anesthesia [20]. Further research is needed to explore strategies for mitigating these adverse effects in clinical practice.

Our analysis of heart rate (HR), mean arterial pressure (MAP), and oxygen saturation (SpO2) during the procedures revealed notable intra-procedural changes. HR values were significantly lower in the Pro-Dex group and increased in the Keto-Fol group compared to baseline. This suggests that dexmedetomidine may exert a more pronounced bradycardic effect, while ketamine may lead to an increase in HR during the procedure. These findings align with the known cardiovascular effects of these agents [12,21,22]. The observed changes in MAP may be related to the combined effects of propofol-induced vasodilation and the cardiovascular actions of dexmedetomidine and ketamine [23]. The decrease in SpO2 during the procedure was transient and similar across all groups, with subsequent recovery to near-normal levels. This may be attributed to the mild respiratory depression associated with propofol administration [24,25], although not seen in all studies [12,26,27]. The observed changes in HR and MAP may reflect the distinct pharmacological properties of the sedative agents used in the different protocols.

This finding suggests that the sedation protocol employed during ERCP may influence post-procedural recovery and the need for extended hospital stay. Regarding the length of hospital stay, patients who received Propofol alone exhibited a significantly longer duration compared to the Keto-Fol group. It is important to note that the length of hospital stay can be influenced by various factors, including the patient's overall health, the complexity of the procedure, and the presence of any post-procedural complications. However, our study aimed to minimize the influence of confounding factors by balancing patient characteristics and procedure details across the four sedation groups. The shorter hospital stay observed in the Keto-Fol group may be attributed to several factors. First, the combination of Ketamine and Propofol used in this protocol may have provided effective sedation while allowing for a faster recovery compared to other regimens. Ketamine, as a dissociative anesthetic, can induce sedation and analgesia while preserving respiratory drive and protective airway reflexes [28]. This may contribute to a quicker recovery and earlier readiness for discharge. Secondly, the sedation depth achieved with the Keto-Fol protocol may have facilitated smoother post-procedural recovery and reduced the need for prolonged monitoring or intervention. The combination of dissociative and hypnotic effects from Ketamine and Propofol, respectively, might have resulted in a balanced sedation state that optimizes patient comfort and facilitates a faster return to baseline functioning. Nonetheless, careful titration and monitoring are essential to minimize the risk of adverse events associated with ketamine administration.

It is worth noting that the Propofol alone group had higher rates of infection, perforation, and bleeding compared to the other groups, although these differences did not reach statistical significance. These complications may have contributed to the longer hospital stay observed in this group. The mechanisms underlying these potential associations require further investigation in larger studies.

The results of this study indicate that individuals with higher BMI are more likely to experience severe outcomes during ERCP procedures, as previously reported in other studies. This association may be attributed to the increased prevalence of obesity-related comorbidities, such as diabetes, hypertension, and cardiovascular diseases, which can lead to more severe complications during the procedure [29]. Additionally, obese patients may have increased intra-abdominal pressure, which can result in respiratory and cardiovascular complications during sedation and ERCP procedures. A study by Mohamed et al. [30] found that obese patients had a higher incidence of complications during ERCP procedures, including pancreatitis, cholangitis, and bleeding, compared to non-obese patients, and required higher doses of sedation and longer hospital stays. However, our study did not identify any significant associations between BMI and sedation-related adverse events, such as apnea, nausea/vomiting, desaturation, hallucination or excitation, hypotension, or bradycardia. This finding is consistent with the results of a study by Deenadayalu et al. [31]. In contrast, our study found that age did not have a significant impact on sedation effectiveness, sedation-related, or ERCP-related adverse events. These findings suggest that higher BMI is associated with more severe outcomes during ERCP procedures, but further research is necessary to elucidate the underlying mechanisms and develop strategies to mitigate the risks associated with obesity during ERCP procedures.

Although this study presents a significant **strength** in its large cohort of 600 patients, which provides ample statistical power to detect significant differences between the various sedation protocols, it is essential to acknowledge its **limitations**. Firstly, the retrospective nature of the study introduces inherent biases and limitations in data collection, and there is a lack of data on endoscopist satisfaction and amnesia. Prospective randomized controlled trials are required to validate the findings and establish causality. Secondly, the study's single-center design may limit the generalizability of the results. Multi-center studies involving larger patient cohorts from diverse populations are necessary to confirm the effectiveness and safety of the investigated sedation protocols.

## 5 Conclusion

In conclusion, our retrospective study comparing different sedation regimens during ERCP highlights the potential benefits of the Pro-Dex protocol in terms of faster achievement of the desired sedation level, lower propofol consumption, and shorter recovery time. Although differences in adverse events were not significant, there was a trend towards higher rates of specific adverse events in certain groups. Further research, including prospective studies, is necessary to establish the optimal sedation approach for ERCP, taking into account both efficacy and safety outcomes. Overall, the findings of this study provide valuable information on the safety and efficacy of different sedative regimens for ERCP. The results of this study can help clinicians to make informed decisions about the best choice of sedative agent for their patients.

## Ethics approval

This study was conducted according to the guidelines of the Declaration of Helsinki. The Institutional Review Board of Shandong University approved this study (KYLL-2021088) and waived the requirement of informed consent due to the retrospective nature of this study and its observational design.

## Availability of data

The corresponding author can provide access to the datasets used in this study upon a reasonable request.

## Funding

This research was supported by the 2022 Shandong Provincial Medical Association Analgesia and Sedation Anesthesia Optimization Special Project (YXH2022ZX05280).

## Consent for publication

I hereby provide consent for the publication of the manuscript detailed above, including any accompanying images or data contained within the manuscript.

## CRediT authorship contribution statement

**Ning Zhang:** Funding acquisition, Formal analysis, Data curation, Conceptualization. **Guanjun Li:** Writing – review & editing, Writing – original draft, Visualization, Investigation, Funding acquisition, Formal analysis, Data curation, Conceptualization.

## Declaration of competing interest

The authors declare that they have no known competing financial interests or personal relationships that could have appeared to influence the work reported in this paper.
